# Standard experimental paradigm designs and data exclusion practices in cognitive psychology can inadvertently introduce systematic “shadow” biases in participant samples

**DOI:** 10.1186/s41235-023-00520-y

**Published:** 2023-10-21

**Authors:** Emma M. Siritzky, Patrick H. Cox, Sydni M. Nadler, Justin N. Grady, Dwight J. Kravitz, Stephen R. Mitroff

**Affiliations:** 1 Department of Psychological & Brain Sciences, The George Washington University, 2013 H St NW, Washington, DC 20006 USA; 2 Department of Psychological & Brain Sciences, Intelligence Community Postdoctoral Research Fellowship Program, The George Washington University, Washington, DC 20006 USA

**Keywords:** Sampling biases, Cognitive psychology, Data exclusion, Individual differences

## Abstract

Standard cognitive psychology research practices can introduce inadvertent sampling biases that reduce the reliability and generalizability of the findings. Researchers commonly acknowledge and understand that any given study sample is not perfectly generalizable, especially when implementing typical experimental constraints (e.g., limiting recruitment to specific age ranges or to individuals with normal color vision). However, less obvious systematic sampling constraints, referred to here as “shadow” biases, can be unintentionally introduced and can easily go unnoticed. For example, many standard cognitive psychology study designs involve lengthy and tedious experiments with simple, repetitive stimuli. Such testing environments may 1) be aversive to some would-be participants (e.g., those high in certain neurodivergent symptoms) who may self-select not to enroll in such studies, or 2) contribute to participant attrition, both of which reduce the sample’s representativeness. Likewise, standard performance-based data exclusion efforts (e.g., minimum accuracy or response time) or attention checks can systematically remove data from participants from subsets of the population (e.g., those low in conscientiousness). This commentary focuses on the theoretical and practical issues behind these non-obvious and often unacknowledged “shadow” biases, offers a simple illustration with real data as a proof of concept of how applying attention checks can systematically skew latent/hidden variables in the included population, and then discusses the broader implications with suggestions for how to manage and reduce, or at a minimum acknowledge, the problem.

## Significance statement

Cognitive psychology research has great promise to directly impact society at large, but this can only happen if the science is robust, replicates, and generalizes. This commentary highlights how two standard aspects of research processes—the nature of typical study designs and data exclusion criteria—may introduce inadvertent “shadow” sampling biases, which could subsequently undermine the generalizability of research in the field, especially if the biases go unacknowledged. It is argued that standard research practices can unintentionally exclude non-random subsets of the participant population and that such systematic undersampling, if unacknowledged and/or undetected, can hinder appropriate generalization and thus reduce applicability. For example, if study designs inadvertently discourage enrollment from individuals at the tail end of a population distribution (e.g., those high in ADHD symptoms), individual differences effects may be more muted than what is actually reflected in society, thus underestimating the full population variability. This may make it difficult to establish the appropriate connection between basic science and various communities that could otherwise benefit from the work. Similarly, inadvertent sampling biases (which are referred to here as “shadow” biases) that selectively exclude non-random subsets of the available participant pool (e.g., those low in conscientiousness) can negatively impact the development of interventions, training programs, or therapies. If a basic scientific result is based on an unacknowledged or unrealized skew in the sampled population, the proposed resources or interventions based on that result may lack sufficient generalizability. Similarly, such biases can hinder efforts to use basic science to inform personnel selection and assessment procedures in applied settings. The current commentary raises these issues, and others, along with an illustrative data example and suggested remedies.

## Introduction

Cognitive psychology studies can take vastly different forms, employing a wide breadth of methods and experimental design choices; yet, there are commonalities across many studies. For example, traditional vision and attention experiments often required participants to sit in a darkened room, positioned with a chinrest, and they were asked to fixate on a screen as they completed hundreds or thousands of trials over the course of a long experimental session (e.g., Duncan, 1980; Nakayama & Mackeben, 1989). More contemporary experiments may forego chin rests and bite bars, but many maintain the same core demands, wherein study participants are asked to complete a repetitive task for an extended period of time. Such tasks may be difficult for certain groups of participants, and those particular participants may be more likely to fail the minimum performance criteria needed to have their data included in the final analyses. Unfortunately, this can lead to a systematic sampling bias where those who fail to make it through the study are not randomly distributed across the full participant population.

Another commonality across cognitive psychology studies is that it is regular practice to have inclusion criteria based on demographic characteristics that are described at recruitment. For example, potential participants might be informed that to enroll in a particular study, they need to have normal or corrected-to-normal color vision, be within a specified age range, or be right-handed. Since such explicit and easily determined criteria are often included in recruitment materials, would-be participants can self-select not to enroll if they know they do not qualify for the study. Further, the explicit criteria that are included in the recruitment screening process are typically reported in the paper’s methods section and are acknowledged, or assumed, as a potential limitation on the study’s generalizability. However, there are additional ways in which would-be participants may self-select not to enroll in a study that likely never make it into a paper’s methods section and are likely to go undetected and unacknowledged. These systematic influences on participant enrollment can create inadvertent and indirect sampling biases—and it is these sorts of hidden, or “shadow,” biases that are the focus of the current commentary.

**Fig. 1:**
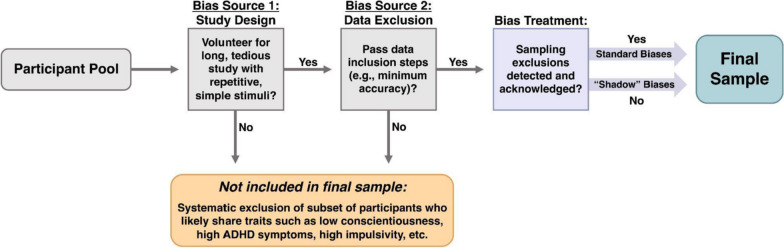
Flowchart of how inadvertent systematic sampling biases in typical cognitive psychology studies can be introduced by two sources—typical study designs and data exclusion practices. Critically, these sampling biases can easily go undetected and unacknowledged by the research team, leaving them as “shadow” biases that may inadvertently impact generalizability

The argument presented here is that the nature of many cognitive psychology tasks—specifically standard recruitment practices and inclusion/exclusion steps—can inadvertently introduce “shadow” biases that affect the generalizability of the results in ways that can easily go undetected and unacknowledged. The goal is to add to existing efforts (e.g., Chandler & Shapiro, 2016; Chandler et al., 2020) that shine light on these inadvertent sources of bias to increase transparency and awareness of the ways in which studies may or may not generalize to various populations. For example, while some individuals will volunteer to participate in, and then successfully complete, cognitive psychology studies, others might find the task aversive and self-select not to participate at all (Fig. 1; Bias Source 1). Likewise, others might agree to enroll in the study but then not achieve the required performance criteria (Fig. 1; Bias Source 2). Each of these steps has the potential to produce systematic sampling biases that can directly, or indirectly, impact generalizability regardless of the experimenters’ intentions.

## Sampling biases via intentional recruitment efforts and samples of convenience

It is inappropriate to make broad claims about society when only examining a specific subset of the population. Early, formal psychology research was riddled with very obvious sampling biases whose limitations have largely been acknowledged (if not at the time of the work, at least more recently). For example, the vast majority of early psychology research was conducted with White male undergraduate students as the participants and with White male researchers conducting the studies (Guthrie, 1998). Interestingly, gender imbalances in psychology participant samples have mostly flipped in recent years; studies that draw participants from undergraduate students enrolled in psychology courses will typically end up with a highly female-skewed participant sample given that ~ 75% to 80% of psychology majors are women (American Psychological Association, 2021). Other sampling biases are pervasive across the field, such as the fact that the majority of research is conducted with Western, Educated, Industrial, Rich, and Democratic (WEIRD) participants (e.g., Henrich et al., 2010). Specific subfields face additional biasing concerns; for example, developmental psychology has thorny sampling issues (e.g., Bornstein et al., 2013) with studies of older adults often conducted with a biased sample of volunteers who have the desire, time, and ability to participate (Murman, 2015). Given the field’s reliance on voluntary research participation (it is not ethical to force or coerce individuals to participate), such limitations are an inevitable and natural part of the research endeavor. Likewise, infant and child research often relies on a guardian being willing and able to voluntarily take the time to bring the child participant to a laboratory setting for the research study, which creates a potential socioeconomic status bias in the participant pool.

While only testing a subset of the broader population is often a limitation, sampling is sometimes intentionally restricted to specific groups for good reasons. If a research team wants to study the impact of nicotine on cognitive performance in nicotine users, they will selectively recruit individuals who regularly use nicotine products (e.g., Swan & Lessov-Schlagger, 2007). Similarly, if researchers wish to explore visual acuity in elite American football players, they very well might selectively recruit professional football players (e.g., Yoo et al., 2010). There are clear and important opportunities in psychology research to intentionally study subsets of the broader population, and much can be gained through such efforts.

In contrast to intentional sampling choices like the examples above, uncontrolled and unacknowledged “shadow” sampling biases that are inadvertently generated can confound and unintentionally limit research conclusions. For example, the majority of research conducted with functional magnetic resonance imaging (fMRI) excludes left-handed participants (Willems et al., 2014). Similarly, Autism research is often done with samples with a higher percentage of male participants, which can systematically diminish the study of females on the Autism spectrum; this bias can contribute to barriers in appropriately diagnosing women with Autism as the diagnostic criteria can be primarily based on male-displayed behaviors and symptoms (e.g., Bruchmüller et al., 2012; Haney, 2016). Such systematic sampling biases can introduce unintended confounds that can distort the conclusions of otherwise sound research. Critically, if such confounds are acknowledged and are part of the discussion of the research, then the research can be appropriately situated in the literature and more accurately generalized to other contexts. However, when such biases go unnoticed and/or unacknowledged, the field as a whole could suffer.

**Fig. 2:**
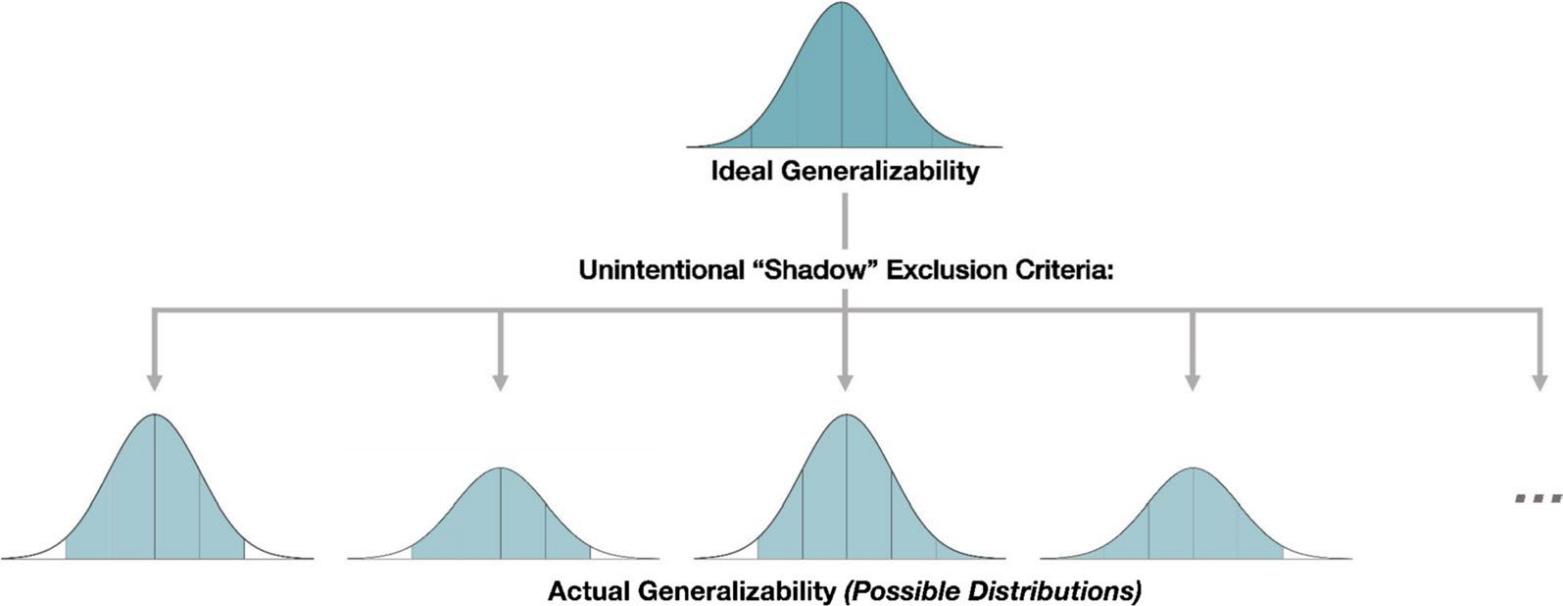
Depiction of sample hypothetical effects on the assumed population distribution resulting from “shadow” sampling biases arising from exclusion criteria that are unintentional and/or unacknowledged (e.g., conscientiousness, ADHD symptomatology)

Some of the above examples involve inclusion and exclusion criteria based on explicitly recognized *demographic* characteristics like handedness and age. Such demographic measures are the most common a priori variables used during the recruitment, analysis, and dissemination of a project that can limit generalizability, and these limitations are often acknowledged, or at least implicitly assumed, by the research team (Fig. 2). For example, results from a study that uses college students as participants would not be assumed to directly generalize to infants. However, there is another key category of individual differences that often goes overlooked—*psychographic* characteristics, such as psychological attributes and personality traits (Demby, 1994; Shaikh et al., 2021). Such psychographic characteristics (e.g., introversion, grit, conscientiousness) can also underlie sampling biases in the same manner as the sources of bias discussed in the examples above, but perhaps in a less obvious way. The next section and the subsequent data example both highlight such hidden “shadow” sources of sampling biases.

## Unintentional sampling biases via recruitment efforts

The above section focuses on explicit recruiting decisions and the natural consequences of using convenience samples, but sampling biases can also inadvertently arise from the nature of the experimental tasks themselves. For instance, cognitive neuroscience research using electroencephalography (EEG) relies on head caps with electrodes that measure the electrical activity on the scalp. However, the caps and electrodes are not designed for coarse and curly hair, which can make it difficult to include Black participants (Etienne et al., 2020). More broadly, the structure of many cognitive psychology study designs may systematically discourage some individuals from enrolling in the studies in the first place. Studies that involve long, repetitive tasks with hundreds of nearly identical trials conducted in a sparse testing space could systematically deter groups of potential participants who are self-aware of their own aversions to such issues, thus introducing inadvertent selection biases. Studies requiring participants to engage with a task for multiple sessions could also unintentionally drive participant attrition as this may implicitly exclude participants who are unable or averse to participating and exercising their attention over a longer time course (e.g., individuals with high levels of ADHD symptoms). Importantly, such inadvertent and unacknowledged sampling biases can artificially reduce, or increase, the likelihood of finding a significant statistical result, which can undermine the goals of the research and the broader implications for society.

## Sampling biases through task demands and attrition

Inadvertent sampling biases can arise from standard data exclusion processes that occur after the enrollment phase. Specifically, researchers regularly establish performance criteria (Fig. 1; Bias Source 2) so that aberrant data are removed from the final analyses. This is an appropriate research practice that is standard in psychology research; it is important to ensure that data analyses are being conducted on appropriate data arising from participants who actually engaged with the task. However, such data cleaning practices can also unintentionally create a sampling bias. As illustrated in Fig. 2, removing data from participants who fail to meet minimum accuracy or speed thresholds could systematically remove certain sections of the broader population from the analyses (and thus from the research project altogether). Similarly, research teams will sometimes include attention checks as inclusion criteria that determine if the participants are appropriately engaged with the task. Attention checks can vary in nature; for example, they can take the form of instructional manipulation checks, “catch trials” that differ from the typical task, or questions used to determine if the participants are engaged with and/or comprehending the task (Abbey & Meloy, 2017; Hauser & Schwarz, 2016; Kane & Barabas, 2019). Participants who fail to correctly engage with the attention checks may have their data removed before the final analyses for a study, ideally preventing an increase in noise or bias created by this lack of attention. When participants do not meet established inclusion criteria, it makes sense to remove their data from the analyses as their data are likely uninformative, skewed, noisy, or otherwise tainted. However, if there is a common thread among the participants who are less likely to meet the inclusion criteria (e.g., those high in ADHD symptoms, those low in conscientiousness), then these standard data cleaning steps can introduce inadvertent sampling biases. Importantly, and as exemplified in the previous section, such introduced biases are *rarely acknowledged or discussed when considering the results of a study*—*making them what we are referring to as “shadow” biases.*

## Inadvertent, unintentional sampling biases example 1: Clark et al., (2015)

To illustrate the above points, consider a previous study (Clark et al., 2015) that produced robust and informative results but may have inadvertently reduced generalizability with its study design. The goal of the Clark et al. (2015) project was to use EEG to gain insight into the nature of learning through practice in visual search. The core question of the study demanded that participants engage in a task long enough to show practice effects. This required participants to stay still in a sterile research environment so that EEG measures could be successfully obtained. Study participants were asked to complete a five-day testing protocol with one hour of testing each day, two of which included EEG measures. During each testing session, the participants completed nearly 2000 trials that involved fixating on a central marker and then making a forced-choice decision about a simple visual display. In this case, the very nature of the research questions (practice effects and using EEG markers) unavoidably asked the participants to engage in a study that required repeated engagement with a tedious task while exerting attentional effort.

From a recruitment standpoint, individuals averse to the long task, the use of EEG, the commitment to enroll in a five-day study, the need to maintain fixation, or any other aspect may have chosen not to participate. In other words, individuals from the extremes of relevant psychographic distributions (e.g., ADHD symptomatology, conscientiousness) may have differentially self-selected into the study, which could have inadvertently excluded subsets of the population in a non-random manner (see Fig. 2). Notably, no such acknowledgment was included in the study discussion or presented as a possible limitation; which is exactly the point being stressed here—such “shadow” biases are easily overlooked and often go unacknowledged.

From a task demands and attrition standpoint, the research team established minimum performance and compliance criteria for a participant’s data to be included in the final analyses. Data from two of the 19 enrolled participants were removed for unreasonably low accuracy in the behavioral task, and another four participants had their data removed for excessive eye and muscle movements during the EEG portion of the task (Clark et al., 2015). It was necessary and appropriate to take such exclusion steps to ensure the final analyses were conducted on clean data with a reduced level of noise; however, might there have been a common, systematic factor contributing to this participant attrition driven by an unacknowledged psychographic characteristic? For example, participants with decreased accuracy and an inability to maintain fixation could, arguably, be high in ADHD symptoms, which could be a non-obvious systematic factor contributing to participant loss in studies requiring sustained attention for either the attention checks or, as seen in this case, the task itself. This notion has some merit given that lab-based tasks can actually be quite similar in nature to clinical tasks used to diagnose ADHD (e.g., continuous performance tests) due to their exacerbation of participants’ ability to sustain attention and inhibit responses (Baggio et al., 2020; Epstein et al., 2003). Research tasks that tax attentional capabilities may disproportionately impact participants who share specific psychographic characteristics (e.g., high in ADHD symptoms), which can create a non-random, systematic underlying driver of attrition that can limit the desired generalizability of a study.

## Inadvertent, unintentional sampling biases example 2: Grady et al. (2022)

For an ongoing research effort in our laboratory (e.g., Grady et al., 2022; Silverman et al., 2022), we ask participants to complete a large collection of approximately 20 self-report surveys during an hour-long testing session and then complete behavioral tasks (e.g., a game-like visual search task with an estimated duration of about 45 min). Participants who we run in-person in our laboratory sign up for two one-hour sessions, and participants who complete the study virtually sign up for a two-hour study that they can engage with at their own pace, but they are generally expected to complete it in one sitting. To be included in the final data analyses, participants must meet several specific inclusion criteria, including passing various attention checks and meeting several specific performance checks on the behavioral tasks (e.g., accuracy and speed thresholds).

In a recent project (Grady et al., 2022), our primary focus was examining individual differences centered around conscientiousness—the goal-directed ability to control impulses, plan, and delay gratification (Roberts et al., 2009). While we had no intention of introducing sampling biases into our project, we now see that multiple steps along the experimental process likely created biases against individuals low in conscientiousness. First, signing up for a multiple-part study is not something everyone will readily agree to. Second, to actually complete the study, the participants had to have some reasonable level of conscientiousness—those tested in the laboratory had to sign up for two different testing sessions and actually attend both, and those tested virtually had to follow explicit instructions, including downloading an app onto their own mobile device and providing the experimenters with specific details from the app. Third, to have their data ultimately included in the final analyses, the participants had to meet our various attention and data quality checks that likely worked to systematically eliminate less conscientious individuals. Notably, we did not acknowledge or discuss such potential biases in our publication (Grady et al., 2022), as this was not an issue we were considering, thus making any effects prime examples of “shadow” sampling biases.

These steps that might have systematically introduced hurdles for those low in conscientiousness are particularly salient for this project given that the primary analyses focused on *an individual differences analysis on conscientiousness*. Our experimental design and data exclusion criteria created a potential bias, albeit inadvertent, that could remove individual variability for the very factor we wished to examine (i.e., systematically removing participants low in conscientiousness). The primary finding of the study was a significant and meaningful influence of conscientiousness on performance (Grady et al., 2022), but it is possible that we created a design that worked against our goals and perhaps dampened the effect.

To demonstrate the systematic biases that can be introduced into analyses through standard experimental practices, we identified participants who failed our criteria for the attention checks introduced into the long series of self-report survey measures. There were three attention checks spread across the surveys, and each was a simple multiple-choice question about the upcoming survey, accompanied by feedback for any wrong answer. If a participant needed four attempts for any one check or seven or more attempts across all three checks, they were deemed an *Excluded* participant based on failing the attention check criterion. Table 1 provides data from 10 of the surveys administered for the Included participants (*N* = 919) and the Excluded participants (*N* = 33). The Included participants are those who did not fail any of the exclusion criteria, and the Excluded participants are those who specifically failed the attention check criterion. Importantly, there are additional participants who did not satisfy all of the inclusion criteria—but the comparison here is between those who passed all criteria and those who failed the attention check step. As can be seen in Table 1, the participants excluded due to failing attention checks differed significantly from those included in terms of their self-reporting of symptoms and qualities for 8 of the 10 surveys evaluated (trait anxiety, ADHD, impulsivity, Autism, OCD, self-control, conscientiousness, and grit). The p values in Table 1 represent Mann–Whitney U tests of significance, given that the assumptions of normalcy were violated; all are 1-tailed tests given the a priori predictions of direction except for the OCD survey, as there was no directional prediction for that measure. This is a subset of available measures but provided here as an example of the kinds of systematic biases that can be inadvertently introduced when applying standard exclusion criteria to a group of participants.

**Table 1:** Data from a subset of surveys completed by a large cohort of participants (see Appendix of Grady et al., 2022 for a full list of surveys) as part of a broader study (e.g., Grady et al., 2022; Silverman et al., 2022)

	Self-report survey	Excluded (*N* = 33) Mean (SD)	Included (*N* = 919) Mean (SD)	Mann–Whitney U value	p value	Rank biserial correlation (effect size)
Predict Excluded> included (1-tailed)	ADHD symptoms	55.33 (24.13)	44.12 (22.87)	19,147	**0.005 **	0.263
	Autism symptoms	21.88 (4.96)	19.04 (5.77)	19,695.5	**0.002**	0.299
	Impulsivity	69.85 (9.82)	62.29 (10.72)	21,296	< **0.001 **	0.404
	Trait anxiety	2.48 (0.30)	2.38 (0.27)	18,374.5	**0.019**	0.212
Predict excluded< included (1-tailed)	Conscientiousness	3.34 (0.51)	3.49 (0.64)	12,460	**0.041**	− 0.178
	Maximization	4.53 (0.82)	4.61 (0.99)	13,885	0.205	− 0.084
	Responsibility	94.94 (23.78)	92.60 (22.00)	16,781	0.851	0.107
	Self-control	111.00 (14.95)	115.94 (19.15)	12,355	**0.035**	− 0.185
No prediction (2-tailed)	CCD symptoms	24.58 (12.94)	16.62 (11.54)	20,866.5	< **0.001**	0.376

Data are separated into those who would be excluded in subsequent analyses based upon failing an “attention check” criterion (“Excluded”) and those that would have passed all inclusion criteria (“Included”). Reported statistics are from Mann–Whitney U tests, and all *p* values are 1-tailed except for OCD, as there was no a priori directional prediction for that survey

Bolded text represents significant values of p < 0.05

## Theoretical implications

The seemingly innocuous research practices discussed here—standard cognitive psychology study designs and data exclusion criteria—can have real, and potentially detrimental, impacts on the generalizability of the science. Academic researchers go through painstaking efforts to design, conduct, and analyze research studies to avoid confounds, but it seems that some of these very efforts may in themselves introduce systematic sampling biases that result in undetected and/or unacknowledged limitations.

For research projects focused on individual differences, the issues raised here have the potential to create a systematic sampling bias wherein specific subsets of the population distribution can be under-sampled. Critically, if the measure of interest (i.e., the specific individual difference metric being explored) is not completely orthogonal from the inadvertent sampling biases, then the science can be impacted. As Table 1 demonstrates, there is the potential for a wide variety of individual differences to be impacted, even if done so unintentionally. This can include psychographic characteristics that could impact a would-be participant’s ability and willingness to volunteer for a study in the first place as well as a participant’s ability to eventually complete the study in a satisfactory manner. Moreover, standard performance-based exclusion criteria can (and likely often do) alter the distribution of participants that make it to the final dataset. Beyond creating skewed samples, these standard practices can even cause individual differences research projects to start at a disadvantage as they are artificially removing natural variability by the likely exclusion of more extreme individuals, which may weaken potentially robust findings.

Unfortunately, non-individual differences research projects are not protected from the detrimental impact of these inadvertent sampling bias problems, as the data are less generalizable. It is difficult to make claims about general tendencies when removing real variability that results from the study being based on a subset of the planned testing population. The impact is compounded when the biases are undetected and unacknowledged.

Finally, the sampling bias concerns discussed here likely contribute to the ongoing “replication crisis” in psychology (Maxwell et al., 2015; Open Science Collaboration, 2015). Replication depends on two factors: effects and variance. Even assuming equivalent effects across replications, samples with differing sampling bias (e.g., a more inclusive participant pool) will have different variance and potentially overestimated effect sizes (Klein et al., 2018). If papers are not fully transparent and fail to report their recruitment practices and inclusion/exclusion criteria (e.g., Bakker & Wickerts, 2014), then replications can be impacted as different studies can have different unintentional “shadow” biases generated by what participant subsets they happen to systematically remove.

## Applied implications

Cognitive psychology research is conducted for a number of reasons, and one core goal of a large number of studies is to inform theories and mechanisms of cognitive processing. In turn, those theories and revealed mechanisms can be leveraged to inform a wide breadth of applied questions with societal impact. One immediate implication of the issues raised here is that unacknowledged “shadow” sources of reduced generalizability can limit the reach of basic psychological research. This can manifest in multiple ways. First, if a study design discourages enrollment from individuals at the extreme ends of a population distribution (e.g., those low in conscientiousness; those high in ADHD symptoms), then any effects of individual differences measured in this subset of the population could be more constrained than what is actually reflected in society. This can lead to a failure to make the appropriate connection between the basic science and various communities that could otherwise benefit from the work. Second, if a study design creates a more constrained set of participants than is assumed or acknowledged (Fig. 2), then future researchers may falsely assume the work is of higher generalizability. In the worst-case scenario, the basic science result may ultimately fail to manifest at the same level of robustness in practice, leading to a loss of confidence in the usefulness of basic science for applied questions. Third, if study designs necessarily discourage some groups of individuals from enrolling, then it is possible that this can create a non-random selection of the broader community that is excluded from the research process. These individuals will not be able to contribute to the science, benefit from the findings that may fail to generalize to them, or directly learn from the science by being a part of the full process.

Individual differences research is a prime area that can be adversely affected by the inadvertent bias effects discussed here, and this raises concerns about how well the results can be applied more broadly. Critically, the exclusion of data from individuals at the extremes of a population distribution could hinder the development of directed interventions, training programs, or therapies that were intended to address the needs of these exact groups of individuals. If the basic science is based on a skewed population distribution that selectively (but without acknowledgment) excludes input from one or both extreme ends, the proposed resources or interventions may lack sufficient generalizability, failing to address the needs of the full range of individuals within the target population. While not all cognitive psychology research aims to directly inform applied or intervention-based work, transparency and sample representation, or a lack thereof, in theoretical work on individual differences could still indirectly affect these groups. Similarly, cognitive psychology research has played a fundamental role in establishing personnel selection and assessment procedures in a number of different industries. If the underlying, basic science is selectively missing part of the potential population—and this is not realized and/or acknowledged—then this enterprise can be significantly hindered.

## Proposed steps to reduce the introduction of unintended sampling biases

Standard experimental design practices and exclusion criteria are an appropriate and necessary part of research, and these steps serve to provide clean data from an appropriate set of participants. When these steps are done explicitly and acknowledged, they generate a participant sample that likely does not fully generalize to the broader population, and this caveat is accepted as part of the research effect. However, these standard practices can also introduce inadvertent “shadow” biases that can be overlooked and hard to address. While there is no easy way to completely avoid or remove sampling biases that are introduced by experimental designs and/or exclusion criteria, there may be steps researchers can take to dampen the issues. One step is to use research designs that provide the participants with more flexibility and agency in how they participate. For example, recent advances in crowd-sourced behavioral data collection have made it possible to efficiently collect large datasets through online participant forums such as Amazon Mechanical Turk (e.g., Paolacci et al., 2010), mobile apps created specifically for research purposes (e.g., Sea Hero Quest, www.seaheroquest.com; Coutrot et al., 2019), community websites designed to crowdsource data from volunteers (e.g., Test My Brain, www.testmybrain.org; Fortenbaugh et al., 2015; Halberda et al., 2012; and Project Implicit, www.projectimplicit.net; Nosek et al., 2002), and data accumulated via mobile games (e.g., Airport Scanner, www.airportscannergame.com; Mitroff et al., 2015; Mitroff & Sharpe, 2017). In contrast to typical in-laboratory studies conducted on college campuses, online data collection (e.g., Amazon Mechanical Turk) allows researchers to tap into population samples that are often significantly more diverse in a number of factors including age and race/ethnicity (Difallah et al., 2018; Weigold et al., 2021). These relatively new ways to collect data also afford some flexibility where participants can contribute on their own terms (e.g., taking breaks, not having to leave their home), potentially expanding who can participate in research studies by giving participants more agency in how they participate. Of course, gathering data through such outlets can introduce other biases and confounds, and there is no single panacea that does not require complementary methodologies. For example, online participant populations may be skewed toward certain personalities who would choose to engage in such environments or may exclude individuals with limited access to internet connections or appropriate devices.

Along the same lines, inadvertent biases in sampling can potentially be partially alleviated by modifying data collection efforts to be more inclusive. Many cognitive psychology studies are prohibitive to certain populations, as not everyone can participate in a study that is over an hour long and demands sustained attention to a tedious and repetitive task. When possible, researchers could run experimental designs that rely on more participants but with fewer trials per participant. Again, online forums may be a useful manner to collect such datasets. Similarly, research teams can be careful to avoid practices that could exacerbate potential biases; for example, being mindful of recruitment of student populations in relation to the timing in the academic term and reporting those details in the methods section of papers (Porfido et al., 2020). Virtual testing also allows participants to do the task on their own time, at their own pace, and in their preferred setting. This is a built-in aspect of online forums such as Mechanical Turk, but researchers can also do virtual testing with on-campus participants to increase accessibility. Students can engage through a virtual format rather than going to a research laboratory, and this can allow them to start and stop, take breaks, and participate at any time of the day. We would also argue that, for experimental studies that can allow it, experimenters should encourage participants to take breaks. Further, individual differences in time perception (e.g., impaired time perception and executive function in ADHD) may unintentionally exclude those who have difficulties with planning and scheduling testing sessions (Ptacek et al., 2019). As such, online data collection methods that allow for impromptu participation could combat issues of attrition by eliminating the need to schedule in-person laboratory visits.

Of course, any of the above suggestions can change the nature of the experimental design, which can introduce different inadvertent biases. For example, the spacing and timing of trials can impact performance, and changing such design aspects could impact task demands. Furthermore, trial learning can vary over different timescales, and some studies may specifically want to focus on the impact of fatigue. There is no singular solution that avoids all potential biases, but it is important to consider ways to minimize or dampen some. When possible, running a study with multiple complementary methods (e.g., both online with few trials per participant and in-person with many trials per participant) may be an ideal way to address such concerns.

Finally, when a study must be done in a more traditional way (e.g., an EEG study where a participant is in a laboratory environment for more than an hour doing the same task hundreds of times or an experimental design that requires all trials are done in a single lengthy session), then the researchers should understand and acknowledge that this is a biased participant population. The hope of the current commentary is to increase awareness of these forms of biases to help reduce instances where “shadow” biases are inadvertently included and not acknowledged, with full awareness that the proper design of many studies necessitates the inclusion criteria and exclusion thresholds that are the source of these shadow biases. If participants must be excluded from the final analyses, researchers could conduct a sensitivity analysis both with and without the Excluded participants to determine the impact of their explicit inclusion and exclusion criteria. It may also be possible to help account for the inadvertent biases by running an accompanying study that gets around the biases to bolster the results (e.g., conduct a complementary online version of the study that replicates key parts of the findings). Regardless, it would be best if the field were to, at a minimum, acknowledge and note the implications for generalizability. Finally, it is in the best interest of the field if researchers adopt open science practices (e.g., data sharing) and are fully transparent about their design, recruiting, methods, and analysis steps. This way, it may be possible to identify possible limitations that could have an impact on sampling biases—even if the original research team *did not realize* the potential issues.

## Conclusions

Common research practices that provide appropriate experimental control in contemporary cognitive psychology studies, such as experimental design choices and explicit exclusion criteria, can inadvertently undermine the generalizability of the very data they are designed to optimize. While these practices are often necessary to obtain sufficient data and to narrow the scope of a sample to address the goals of the study in question, it is important to acknowledge the potential introduction of such subtle and unintended limitations to the generalizability and replicability of the findings. In line with many explicit steps in psychology to increase transparency, the current commentary highlights the need for recognizing, and then acknowledging, the various forms and sources of limitations in the research practices.

## Data Availability

The data for the empirical example provided in the current manuscript are from Grady et al., 2022 and available on the project’s OSF site (https://osf.io/8jzwc/). Additional data related to the Excluded participants are available upon request.
